# Autotaxin-Lysophosphatidic Acid: From Inflammation to Cancer Development

**DOI:** 10.1155/2017/9173090

**Published:** 2017-12-21

**Authors:** Silvia Anahi Valdés-Rives, Aliesha González-Arenas

**Affiliations:** Departamento de Medicina Genómica y Toxicología Ambiental, Instituto de Investigaciones Biomédicas, Universidad Nacional Autónoma de México, Ciudad Universitaria, Coyoacán, 04510 Ciudad de México, Mexico

## Abstract

Lysophosphatidic acid (LPA) is a ubiquitous lysophospholipid and one of the main membrane-derived lipid signaling molecules. LPA acts as an autocrine/paracrine messenger through at least six G protein-coupled receptors (GPCRs), known as LPA_1–6_, to induce various cellular processes including wound healing, differentiation, proliferation, migration, and survival. LPA receptors and autotaxin (ATX), a secreted phosphodiesterase that produces this phospholipid, are overexpressed in many cancers and impact several features of the disease, including cancer-related inflammation, development, and progression. Many ongoing studies aim to understand ATX-LPA axis signaling in cancer and its potential as a therapeutic target. In this review, we discuss the evidence linking LPA signaling to cancer-related inflammation and its impact on cancer progression.

## 1. Introduction

Lysophosphatidic acid (LPA) consists of an acyl chain at the sn-1 (or sn-2) position of a glycerol backbone and a phosphate head group. It is the smallest (molecular weight: 430–480 Da) and the simplest bioactive glycerophospholipid derived from membrane phospholipids [1, 2]. Nevertheless, it is involved in a wide range of activities, from phospholipid synthesis to a number of physiological responses as a lipid mediator [3]. LPA activates at least six G-coupled protein receptors (LPA_1–6_) stimulating different signaling pathways through heterotrimeric G proteins such as G_i/0_, G_12/13_, G_q/11_, and G_s_. The outcome of LPA signaling is dependent on cellular context and impacts on biological processes such as wound healing, differentiation, neurogenesis, and survival, to name a few [4]. Due to its small structure, LPA is water soluble and concentrations > 5 *μ*M have been reported in serum; concentrations < 1 *μ*M have been found in other biofluids such as plasma, saliva, follicular fluid, cerebrospinal fluid, and malignant effusions [5–7]. It is known that ATX-LPA signaling increases during wound healing, and both are produced and detected in blister fluids, where they mediate platelet aggregation and skin reepithelization [8]. During this process, ATX-LPA signaling induces production of proinflammatory cytokines. Therefore, aberrant activation of this axis promotes an inappropriate immune response that leads to a proinflammatory state in pathologies like cancer [9].

## 2. Lysophosphatidic Acid Synthesis and Metabolism

LPA is a membrane-derived lysophospholipid from phosphatidylcholine (PC), phosphatidylserine (PS), and phosphatidylethanolamine (PE) [7]. Therefore, several species can be found, differing only in the length and saturation of the acyl or alkyl fatty acid chain [7, 10]. The most abundant plasma LPA species are 18:2 > 18:1 ≥ 18:0 > 16:0 > 20:4 with an acyl group [11, 12]. Although acyl-LPA 18:2 is the most numerous species, acyl-LPA 18:1 is the most frequently used in current research [13].

**Figure 1:**
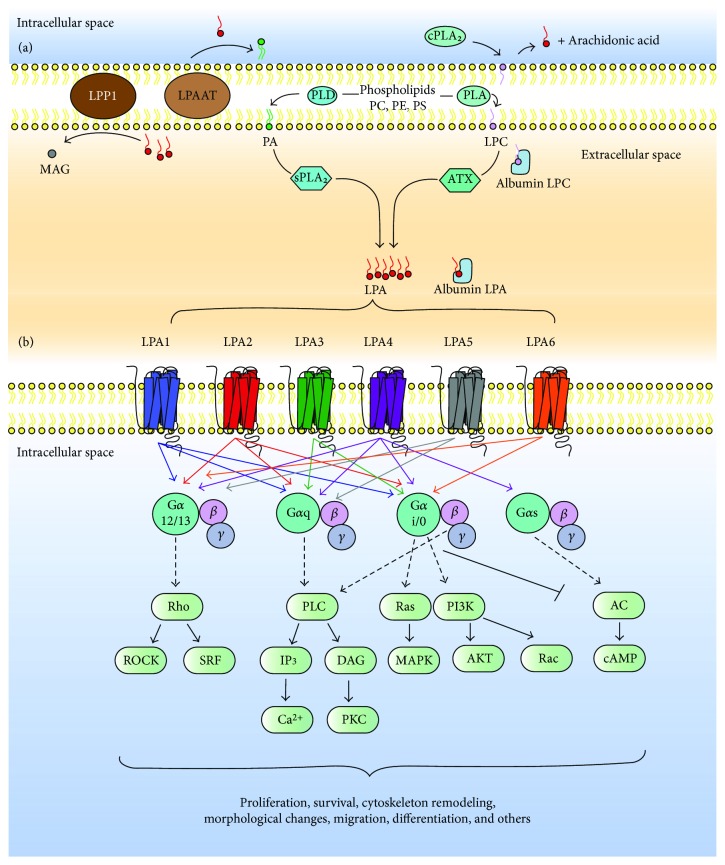
LPA production, metabolism, and signaling. (a) LPA species are derived from membrane phospholipids. PLA removes a fatty acid chain from PC, PE, or PS converting them into lysophospholipids. Afterwards, ATX removes the head group from LPC < LPE < LPS and produces LPA. LPC can derive from cell membrane or circulating LPC bound to albumin. LPA can also be produced intracellularly by cPLA2 from LPC producing LPA and arachidonic acid. On the other hand, PLD can remove the head group from membrane phospholipids and produce PA. Then, sPLA2 removes a fatty acid chain producing LPA. Two enzymes metabolize LPA, LPP1 in the outer leaflet of the membrane hydrolyzes LPA into MAG, and LPAAT transfers an acyl chain to LPA in the inner leaflet of the membrane producing PA. (b) LPA signals through at least six GPCRS (LPA_1–6_) that couple to different G*α* proteins to elicit activation of Rho, PLC, Ras, PI3K, and adenylyl cyclase (AC) and mediate diverse processes that are cell and context dependent. This figure is reproduced from Blaho and Hla [29] (under the Creative Commons Attribution License/public domain).

There are two major pathways for LPA production (Figure 1(a)). The main pathway is the cleavage of membrane phospholipids into lysophospholipids by the removal of a fatty acid chain by phospholipase A (PLA1 or PLA2). Subsequently, ATX cleaves the head group (choline, ethanolamine, or serine) on the lysophospholipids and turns them into LPA [14]. ATX (also known as ENPP2) is a 125 kDa-secreted enzyme from the family of ectonucleotide pyrophosphatases/phosphodiesterases (reviewed by [15]) located on Chr8q24 [16]. Among the seven members of this family, ATX is a unique enzyme that shows lysophospholipase D activity [17, 18]. This enzyme produces most of the extracellular LPA. *Enpp2^+/−^* mice and inhibitors targeting ATX decrease LPA plasma concentration by >50% [19–22]. ATX generates LPA from plasma membrane phospholipids and from circulating lysophosphatidylcholine (LPC) bound to albumin [23]. ATX is essential for development since *Enpp2^−/−^* is lethal at embryonic day 9.5–10.5, with marked vascular and neural tube defects [20, 21]. ATX is also important in adipogenesis since it is upregulated during preadipocyte differentiation to adipocytes and secreted into circulation by the adipose tissue [24].

A second, less common, route of LPA production is the cleavage of phospholipids into phosphatidic acid (PA) by phospholipase D (PLD) at the cell surface. PA is then hydrolyzed in the outer leaflet of the plasma membrane by secreted PLA2 (sPLA2) releasing LPA to the microenvironment [15].

LPA turns over with a half-life of about 3 min in the circulation [25]. Therefore, its main effects are autocrine and paracrine when bound to albumin [10]. LPA turnover is regulated by ATX activity and LPA degradation by lipid phosphate phosphohydrolase type 1 (LPP1) which hydrolyze LPA into monoacylglycerol (MAG) in the outer leaflet of the cell membrane [26, 27] and LPA-acyltransferase (LPAAT), which transfer an acyl chain to LPA converting it into PA in the inner leaflet of the cell membrane [10]. Recently, a negative feedback loop has been described for the ATX-LPA axis [28]; in this mechanism, LPA signaling through its receptor LPA_1_/_3_ induces downregulation of ATX mRNA. Similarly, low levels of circulating LPA increase ATX mRNA, particularly in the adipose tissue of female Balb/c mice [28].

## 3. LPA Receptors

**Figure 2:**
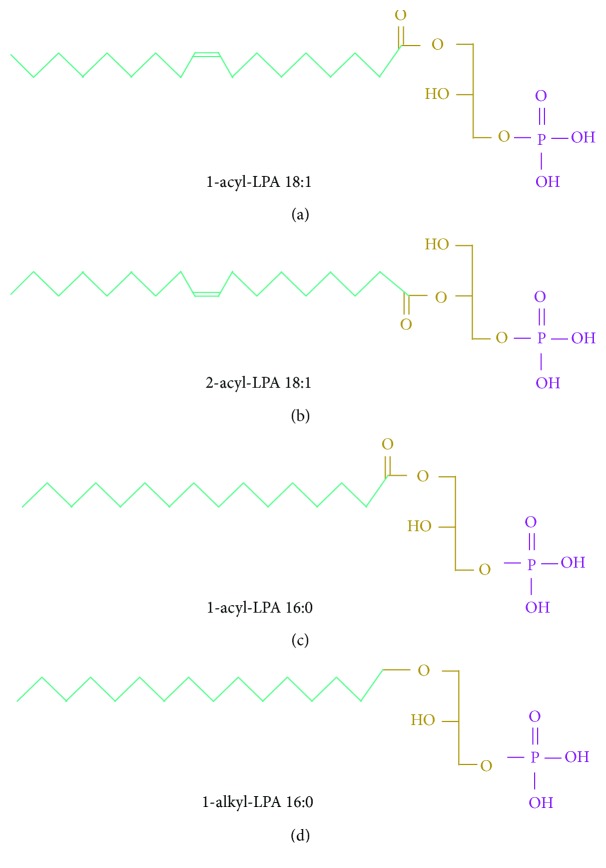
LPA species. LPA is derived from phospholipids with different lengths and saturations. (a) 18 carbon LPA species with an acyl group in sn-1 position and one saturation are the most potent activator of the LPA_1_ and LPA_2_ receptors [7]. (b) Acyl LPA with 18 carbons, one saturation, and the fatty acid chain in sn-2 position are the most potent activator of LPA_3_ and LPA_6_ [2, 34]. (c) An alkyl-LPA species with 16 carbons and no saturation are the most potent activator of LPA_5_ receptor [33].

As previously mentioned, LPA signals through at least six G protein-coupled receptors LPA_1–6_ (Figure 1(b)): gene names are *LPAR1-LPAR6* (human) and *Lpar1-Lpar6* (mouse) [30, 31]. All LPA receptors are rhodopsin-like, with seven transmembrane domain receptors that range from 39 to 42 kDa and differ in their tissue distribution and downstream effectors [7]. According to their homology, there are two LPA receptor families: the endothelial differentiation gene (EDG) family and the non-EDG family [32, 33]. In addition to homology, they differ in their activation by different LPA species (Figure 2). Although acyl-LPA 18:2 is the most abundant species, the EDG family is more potently stimulated by acyl-LPA (LPA_1/2_), and LPA_3_ preferentially bounds to 2-acyl-LPA. The non-EGD family member LPA_5_ is more potently stimulated by alkyl-LPA and LPA_6_ by 2-acyl-LPA, specifically [33]. These differences show that a wide range of physiological effects is modulated through these receptors and LPA species in a context and cell type-dependent manner.

### 3.1. Endothelial Differentiation Gene Family

In 1996, LPA_1_ was the first receptor to be identified and it is the best studied to date. Hecht et al. [35] described a neuroblast cell line overexpressing the ventricular zone gene-1 receptor (Vgz-1), to which LPA binds specifically to induce cell rounding and activation of G*α*_i_. Also known as EDG-2, Vgz-1 was later renamed LPA_1_. Right after its discovery, two other orphan receptors, LPA_2_ and LPA_3_, were identified based on their homology to LPA_1_ [36–38].

LPA_1_ is a 41 kDa protein of 364 amino acids located in Chr9q31.3 and consists of at least 5 exons [30, 31]. This receptor couples with and activates 3 types of G protein, G*α*_i/0_, G*α*_q/11_, and G*α*_12/13_, which initiate downstream signaling through PI3K/AKT, Rho, MAPK, and PLC (Figure 1(b)). These pathways are involved in several cellular processes, including cell proliferation and survival, adhesion, migration, AC inhibition, and Ca^2+^ mobilization [31, 39]. It is widely expressed in most tissues such as brain, uterus, testis, lung, small intestine, heart, stomach, kidney, spleen, thymus, and skeletal muscle at different developmental stages with a variable expression, particularly in the central nervous system (CNS) [36, 39], where, during development, LPA_1_ is found in the ventricular zone, superficial marginal zone, and meninges. After birth, LPA_1_ expression is reduced in the aforementioned areas and continues in oligodendrocytes, particularly during myelination, as well as in astrocytes, where it elicits a wide range of processes (reviewed by [40]). Targeted deletion of *Lpar1^−/−^* showed a 50% of perinatal lethality related to an impaired suckling behavior probably due to defective olfaction. Surviving mice showed craniofacial malformations and reduced body size [41]. Additionally, LPA_1_ has been closely related to the induction of neuropathic pain due to nerve injury via LPA_1_/RhoA/rock-mediated demyelination with a subsequent loss of the structural and functional integrity of neurons, as discussed elsewhere [42].

LPA_2_ receptor (EDG-4) has a ~50–60% homology to LPA_1_, with an estimated mass of 39 kDa and 348 amino acids [36]. Located on Chr19p12, it consists of 3 exons in both humans and mice [30, 39]. LPA_2_ couples to the same G proteins as LPA_1_ (Figure 1(b)): G*α*_i/0_, G*α*_q/11_, and G*α*_12/13_ [36, 39]; therefore, it can similarly activate downstream signaling but, unlike LPA_1_, can also promote migration through the focal adhesion molecule TRIP6 [43, 44]. LPA_2_ activation is associated with survival and migration. Compared with LPA_1_, its expression is more diffuse during development, more restricted in adults, and with high expression in leukocytes and testis in humans and in kidney, uterus, and testis in mice [36, 39, 45]. LPA_2_ knockout mice are mostly normal, suggesting a possible functional redundancy in relation to LPA_1_. A *Lpar1^−/−^ and Lpar2^−/−^* model has also been evaluated [46]. In this model, *Lpar1^−/−^* phenotype predominated with 50% perinatal lethality, cranial malformations, and reduced body size, but it also exhibited frontal hematomas [46].

LPA_3_ receptor (EDG-7) contains 3 exons, has 353 amino acids, and a 40 kDa-estimated mass [37, 38]. This receptor has 52% and 48% homology with LPA_1_ and LPA_2_, respectively, and is located on Chr1p22.3-p31.1 [30, 38, 39]. LPA_3_ couples to G proteins, G*α*_i/0_ and G*α*_11/q_ (Figure 1(b)), and therefore mediates downstream activation of MAPK, PLC, and inactivation of AC [47]. It has been reported that this receptor is more potently activated by 2-acyl-LPA with unsaturated fatty acids [2]. In humans, LPA_3_ is expressed in heart, lung, pancreas, prostate, testis, ovaries, and brain [37]. In mice, it is expressed in testis, kidney, lung, intestine, and moderately, small intestine [39]. Functional deletion of LPA_3_ in female mice showed delayed and defective embryo implantation through the downregulation of cyclooxygenase-2 (COX-2) and reduced levels of prostaglandins, which are essential for this process [48]. In deficient LPA_1–3_ male mice, an independent of testosterone signaling reduced sperm count and mating activity was found [49]. This evidence suggests the role of LPA_3_ in reproductive functions.

### 3.2. Nonendothelial Differentiation Gene Family

In 2003, the first LPA receptors structurally distant from the EDG receptor family were described [50]. The orphan GPCR P2Y9/GPR23 has only 20–24% homology with LPA_1–3_, but it specifically binds to LPA. Its signaling promotes an increase in intracellular Ca^2+^ concentration and adenylyl cyclase activity in “LPA receptor-null” cells exogenously expressing P2Y9 [50]. Soon, LPA_5_ and LPA_6_ description followed [51–55].

LPA_4_ (P2Y9/GPR23) is encoded by 1 exon containing 370 amino acids with a 42 kDa mass [30, 50, 56]. Located on ChrXq21.1, it was the first to be described that couples to four G proteins: G*α*_i/0_, G*α*_11/q_, G*α*_12/13,_ and G*α*_s_ (Figure 1(b)) [57]. LPA_4_ signaling promotes Rho-mediated neurite retraction and stresses fiber formation, Ca^2+^ mobilization, and regulation of cAMP concentration [57]. In humans, LPA_4_ expression is high in ovaries, moderated in thymus and pancreas, and low in brain, heart, small intestine, testis, prostate, colon, and spleen [13, 50]. In mice, it is expressed in heart, ovaries, thymus, skin, and developing brain [57, 58]. *Lpar4 *^−/−^ mice showed no apparent abnormality, but there was a 30% lethality, probably due to blood vessel defects during embryogenesis [58, 59].

LPA_5_ (GPR92) is a 41 kDa protein consisting of 372 amino acids coded in an intronless open reading frame [51, 52]. This receptor is located on Chr12p13.31 and has a 35% homology with LPA_4_ [51, 52]. LPA_5_ couples to G proteins, G*α*_11/q_ and G*α*_12/13_ (Figure 1(b)), by which Ca^2+^ mobilization, inositol phosphate production, neurite retraction, and stress fiber formation are mediated [51, 52]. It has been reported that LPA_5_ preferentially binds to alkyl-LPA (16:0), rather than acyl-LPA (18:1) [33]. LPA_5_ is found in heart, placenta, spleen, brain, lung, and gut in humans [51]. It is also highly expressed in the lymphocyte compartment of the gastrointestinal tract and platelets [51, 60]. In mice, it is found in the brain, heart, kidney, liver, lung, muscle, skin, spleen, stomach, small intestine, testis, and thymus [52]. *Lpar5^−/−^* mice have no apparent phenotypic defects but show a reduced pain sensitivity, faster recovery from inflammation, and reduction in social exploration [61, 62]. They also exhibit nocturnal hyperactivity and anxiety compared to *Lpar5^+/+^* mice [61]. Null mice were also protected from developing neuropathic pain by a mechanism different from LPA_1_ [62].

LPA_6_ (P2Y5) is the most recently identified LPA receptor and the last accepted by the IUPHAR Nomenclature Committee in 2010 [31, 53, 54]. It is a 344-amino acid protein with an estimated mass of 39 kDa [30]. Regarding homology with LPA_4_ [50], it is the closest receptor and is located on Chr13q14 [30, 55]. LPA_6_ couples to G*α*_i/0_ and G*α*_12/13_ (Figure 1(b)), by which a decrease in cAMP, Rho-dependent morphological changes, Ca^2+^ mobilization, and MAPK activation are mediated [53, 54]. It has also been reported that LPA_6_ is preferentially activated by 2-acyl-LPA, rather than 1-acyl-LPA [53]. This receptor has been found in rats' brain, heart, lung, kidney, pancreas, liver, stomach, and small and large intestine [54]. In humans, it has been related to hair growth since a mutation of *LPAR6* was found in patients with hypotrichosis simplex, an alopecia-causing disorder [55].

### 3.3. EDG and Non-EDG Receptor Effects in Cancer

Extensive evidence demonstrate that the receptors from the EDG family promote tumor progression in a wide variety of cancers by enhancing proliferation, survival, migration, and invasion [7]. Conversely, evidence shows that members from the non-EDG family have the opposite effect.

Reconstitution of *Lpar4* in mouse embryonic fibroblasts derived from *Lpar4^−/−^* mice reduces cell motility due to an LPA-induced decrease in Rac activation [58]. Also, LPA_4_ expression in colon cancer cells (DLD1 and HTC116) suppresses cell migration and invasion compared to null-LPA_4_ cells [58, 63]. Similarly, in rat sarcoma cells, overexpression of *Lpar5* significantly reduced motility and suppressed MMP2 activation. On the other hand, *Lpar5* knockdown induced the opposite effect [64]. In B16F10 mice melanoma cells, LPA_5_ reduced migration through a cAMP/PKA-dependent pathway and induced chemorepulsion instead of attraction via LPA [65]. Additionally, in colon cancer cells, lines DLD1, and HCT116, LPA_6_ expression significantly reduced cell growth and motility [63].

In rat lung adenocarcinoma, loss of LPA_3_ due to methylation of the promoter enhances tumor progression by increasing invasion, suggesting a protective role of LPA_3_ in this neoplasia [66]. By contrast, in human fibrosarcoma, LPA_4_ was shown to increase cAMP levels and subsequently activate Rac1 to induce invadopodia, a process directly correlated with invasion and metastasis [67]. Additionally, in rat lung carcinoma, LPA_5_ is highly expressed due to unmethylation of the promoter, and cells expressing only LPA_5_ showed enhanced proliferation, migration, and invasion [68]. Moreover, hepatocellular carcinoma (HCC) cells overexpressing LPA_6_ sustain an increase in tumor growth, migration, and invasion. Moreover, LPA_6_ expression was associated with a worse clinical outcome in these patients [69].

In brief, LPA receptors can have homologous and antagonistic effects depending on the tumor. Therefore, they should be studied in a cancer-specific context to better evaluate their role in tumor development and progression, as well as their potential therapeutic value.

## 4. Autotaxin-LPA Axis in Cancer-Related Inflammation

Since the 19th century, an association between inflammation and cancer was proposed [70]. Inflammatory components are often present in most types of cancer, such as white blood cells, tumor-associated macrophages, and proinflammatory ILs [70, 71]. In several cases, inflammation can predispose individuals to certain types of cancer, including cervical, gastric, colon, hepatic, breast, lung, ovarian, prostate, and thyroid cancer [72–81]. There is also evidence that the use of nonsteroidal anti-inflammatory drugs can reduce the risk of developing colon and breast cancer and reduce the related mortality, as discussed elsewhere [82, 83].

In general, two mechanisms have been proposed to link inflammation and cancer. In the intrinsic pathway, genetic events promoting development initiate the expression of inflammation-related circuits leading to an inflammatory microenvironment. Conversely, in the extrinsic pathway, inflammatory conditions facilitate cancer development. In both cases, a cancer-related inflammation (CRI) is induced and it is proposed as a tumor-enabling characteristic and the seventh hallmark of cancer [71]. CRI enables unlimited replicative potential, independence of growth factors, resistance to growth inhibition, escape of cell death, enhanced angiogenesis, tumor extravasation, and metastasis [84]. Therefore, understanding key components of inflammation is important for better therapeutics in cancer and other diseases.

The ATX-LPA axis is involved in wound healing response, where it induces platelet aggregation, lymphocyte homing, cytokine production, keratinocyte migration, proliferation, and differentiation under physiological conditions [85]. When acute inflammation becomes chronic in unpaired homeostasis, ATX-LPA signaling induces an augmented cytokine production and lymphocyte infiltration, aggravating the inflammation in conditions such as asthma, pulmonary fibrosis, and rheumatoid arthritis, to name a few [86]. In a cancer context, it also promotes cell survival, proliferation, migration, invasion, and angiogenesis, enhancing its progression in a state similar to a “wound that never heals” [84, 87].

### 4.1. Lung

ATX-LPA axis has been studied in airway inflammation where protein kinase C *δ* (PKC*δ*) mediates LPA-induced NF*κ*B transcription and IL-8 secretion in human bronchial epithelial cells (HBEpCs) [88]; LPA activation of PKC*δ*/NF*κ*B and IL-8 production were inhibited by rottlerin (a nonspecific PKC*δ* inhibitor) and by an overexpression of dominant-negative PKC*δ*. *In vivo* LPA administration in mice leads to increased levels of a murine homolog of IL-8 and of neutrophils in the bronchoalveolar fluid [88]. Moreover, LPA signaling induces EGFR transactivation via Lyn kinase, from Src kinase family, to promote matrix metalloprotease (MMP) secretion as well as IL-8 [89]. Additionally, activation of the signal transducers and activators of the transcription 3 (STAT3) in alveolar epithelial cells during host defense promotes inflammation and spontaneous lung cancer [90]. Through these signaling cascades, a chronic inflammation is pursued and could lead to malignant transformation. In lung cancer, inhibition of ATX-LPA axis reduced cell migration, invasion, and vascularization in a 3-D lung cancer xenograft model [91]. There is evidence that ATX is highly expressed in poorer differentiated lung carcinomas, particularly in tumor-adjacent B lymphocytes [92] and that LPA_5_ may play a key role in the progression of these carcinomas [68], while LPA_3_ could have a protective role [66]. Furthermore, LPA and other phospholipid levels are upregulated as a side effect of chemo- and radiotherapy, inducing a prometastatic microenvironment in lung cancer [93]. Interestingly, LPA did not induce proliferation nor survival in these cells, but rather an increase in motility, adhesion to bone marrow stroma, and enhanced secretion of ATP, another potent chemokinetic factor, from stroma cells [93]. Together, evidence suggests a significant role of ATX-LPA axis in inflammation and lung cancer through the increase of proinflammatory cytokines.

### 4.2. Breast

In breast cancer (BCa), the ATX-LPA axis induces inflammation and tumor formation in the mammary gland through LPA_1–3_ and high ATX expression, which is produced in the adjacent mammary adipose tissue rather than actual cancer cells [94, 95]. Individual overexpression of each of the EDG family receptors, but especially of LPA_2_, induced a high frequency of late-onset, estrogen receptor (ER) positive, and invasive and metastatic mammary cancer [94]. Moreover, bone metastases are frequent in BCa; ATX expression in these tumors can control the progression of osteolytic bone metastases *in vivo* through the procoagulant activity of BCa cells that induce platelet-derived LPA [96].

ATX-LPA axis is a strong inducer of inflammatory mediators like IL-8, IL-6, TNF-*α*, and growth factors such as the vascular endothelial growth factor (VEGF) and the granulocyte colony-stimulating factor (G-CSF) [95]. Some molecules (IL-8 and VEGF) were detected earlier than tumorigenesis *in vivo* [94]. Inhibition of ATX induced a twofold reduction in at least 20 of these inflammatory mediators in the tumor-adjacent mammary adipose tissue-reducing inflammation and tumorigenesis [95]. Additionally, expression of LPA_1–3_ increased phosphorylation of STAT3, STAT5, NF*κ*B and ATF2, and master inflammatory transcription factors, in mouse mammary carcinomas [94]. Furthermore, cytokines produced in the microenvironment (i.e., IL-6) can activate STAT3 through its receptors inducing an inflammatory loop [97]. Adipose tissue adjacent to breast tumors stimulates autotaxin (ATX) secretion, which increases tumor growth and metastasis [19]. Interestingly, radiotherapy in adipose tissue of rats and humans increased mRNA expression of ATX, multiple inflammatory mediators, and LPA_1–2_. Such effect could promote LPA signaling and further inflammatory signaling, which in turn could potentially protect cancer cells from subsequent radiation therapy [98]. ATX inhibition reduced the leukocyte infiltration and tumor growth *in vivo* [95]. All these evidence suggest that chronic inflammation contributes to tumor development in BCa. Controlling inflammation and cancer progression could be achieved by targeting the ATX-LPA axis.

### 4.3. Ovary

In ovarian cancer (OC), ATX is highly expressed and secreted by cancer cells [99]. Therefore, LPA is present at high concentrations in the ascites fluid of OC patients compared to benign and healthy controls and has been proposed as a potential biomarker [100–102]. LPA acts as a growth factor and prevents apoptosis in OC cells by signaling through redox-dependent activation of ERK, AKT, and NF*κ*B signaling pathways. Inhibiting ROS production blocked LPA/NF*κ*B signaling and cell proliferation [103]. Additionally, LPA has been shown to upregulate the expression of human telomerase reverse transcriptase (hTERT) and telomerase activity in OC cell lines, through a PI3K and HIF-1*α*-dependent mechanism, enabling replicative immortality [104]. On the other hand, OC cell lines, SKOV-3, and OVCAR3 that expressed increased LPA_1–3_ receptors showed more invasiveness compared to knockdowns. Moreover, via LPA_2–3_, OC cells promote production of IL-6, IL-8, and VEGF *in vitro* [105] and induced urokinase plasminogen activator (uPA) secretion in a MAPK- (p38) and PI3K-dependent mechanism that required Src kinase for optimal MAPK phosphorylation, enhancing OC invasion [106].

### 4.4. Liver

Liver cirrhosis, a terminal stage of chronic inflammatory and fibrotic liver diseases, and chronic hepatitis C are distinct risk factors for hepatocellular carcinoma (HCC) [107, 108]. Increased serum ATX activity and plasma LPA levels have been found in patients with chronic hepatitis C in association with a histological stage of liver fibrosis [108]. Furthermore, in HCC, ATX is expressed in 89% of tumor tissues, especially in those with cirrhosis or hepatitis C, compared to 20% in normal hepatocytes [109]. Additionally, in HCC cell lines, TNF-*α*/NF*κ*B pathway, known to contribute to inflammation-associated cancer, was shown to upregulate ATX expression and LPA production. The latter resulted in an increased cellular invasion [109]. Similarly, LPA modulates tumor microenvironment by inducing transdifferentiation of peritumoral fibroblasts to a CAF-like myofibroblastic phenotype which enhances proliferation, migration, and invasion in HCC [110]. Additionally, LPA_6_ mediates tumor growth and tumorigenicity by upregulating Pim-3 protooncogene through a STAT3-dependent mechanism [69]. Recently, human cirrhosis regulatory gene modules were identified through a transcriptome meta-analysis [107]. This analysis provides an overview of a molecular dysregulation common to a wide range of liver disease etiologies in which the ATX-LPA axis is a central regulator [107]. This study marks a great breakthrough in the area and provides a promising target for HCC chemoprevention through this axis; mainly due to the compounds of ongoing clinical trials on idiopathic pulmonary fibrosis and systemic sclerosis (Table 1). If approved, they could be tested as preventive therapy in cirrhosis patients and as adjuvant therapy in HCC [107, 111].

### 4.5. Colon

In human colorectal cancer (CC), expression of LPA_1_ and LPA_2_ is increased compared to normal mucosa. Conversely, LPA_3_ has a low expression in malignant tissues [112]. Evidence suggests a probable role of LPA_1/2_ receptors in CC. Furthermore, LPA-stimulated proliferation through the MAPK pathway, as well as migration through Rho kinase, and chemoresistance through the PI3K/AKT pathway [113]. Inflammation is an established risk for developing CC. Interestingly, in a colitis-associated mice cancer model, *Lpar_2_^−/−^* showed a decrease in tumor incidence and in progression to colon adenocarcinomas by reducing proliferation and proinflammatory factors such as monocyte chemoattractant protein-1 (MCP-1) and macrophage migration inhibitory factor (MIF) [114]. The latter affected the infiltration of macrophages to the tumor microenvironment [114]. Moreover, although LPA increased tumor incidence in Apc^Min/+^ mice predisposed to adenomas, in *Lpar_2_^−/−^* Apc^Min/+^, tumor incidence was reduced by 50% [114, 115]. In addition, the expression levels of KLF5, cyclin D1, c-Myc, and HIF-1*α* were lower compared to Apc^Min/+^ mice, while *β*-catenin was primarily cytoplasmic in *Lpar_2_^−/−^* Apc^Min/+^ mice compared to its nuclear localization in Apc^Min/+^ mice [115]. This evidence suggests an important role of ATX-LPA axis in tumorigenesis derived from colon chronic inflammation.

### 4.6. Others

Along with cancers previously described, ATX-LPA axis and its signaling pathways have been studied in several other carcinomas such as melanoma, where LPA signaling suppresses antigen receptor signaling, cell activation, and proliferation in CD8 T cells that express LPA_5_, inhibiting immune response [116] and promoting tumorigenesis. In pancreatic cancer, LPA_1_ and LPA_3_ promote proliferation, invasion through MMP2 secretion, and activation of focal adhesion kinase (FAK) and Paxillin, as well as drug resistance [117, 118]. In glioblastoma multiforme (GBM), an increased ATX-LPA axis has been described to promote cell proliferation and migration through LPA_1_ [119]. GBM is also characterized by high levels of inflammatory mediators and activation of AKT and NF*κ*B signaling pathways, although the link between ATX-LPA and inflammation remains to be studied [120]. In thyroid cancer, ATX is highly expressed in papillary thyroid carcinomas compared with benign neoplasm [121]. ATX-LPA axis induces at least 16 inflammatory mediators, including IL1-*β*, IL6, IL8, G-CSF, and TNF-*α in vivo*; at the same time, these mediators induce ATX expression and increase LPA levels. Blocking the ATX-LPA axis induced a reduction of inflammatory mediators, tumor volume, and angiogenesis [121]. In renal cell carcinoma, ATX-LPA axis is associated to chemoresistance through LPA_1_. Coadministration of Ki16425, an LPA_1/3_ antagonist, with sunitinib, a tyrosine kinase inhibitor, prolonged the responsiveness of renal cell carcinoma to sunitinib in xenograft models [122].

**Figure 3:**
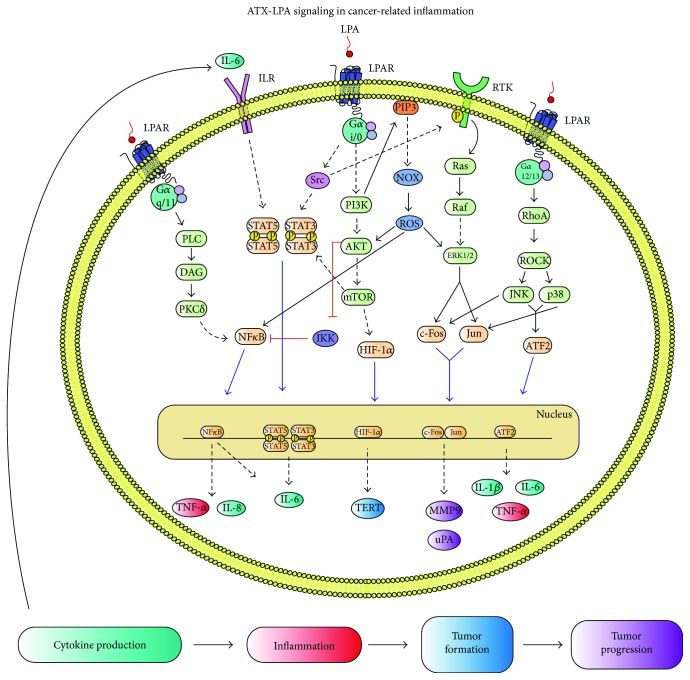
ATX-LPA axis promotes cancer-related inflammation. In CRI, LPA acts on its receptors via G*α*_q/11_, G*α*_i/0_, and G*α*_12/13_. G*α*_q/11_ induces NF*κ*B activation through PKC*δ* promoting TNF-*α*, IL-8, and IL-6 production. G*α*_i/0_ induces the PI3K/AKT/mTOR pathway culminating in NF*κ*B and HIF-1*α* translocation to the nucleus. HIF-1*α* induces the transcription of TERT enabling replicative immortality. G*α*_i/0_ can also transactivate Src kinase and crosstalk with EGFR, to induce extracellular matrix degrading proteins, and STAT-3 signaling pathway to further induce cytokine production. PI3K signaling promotes ROS production and activation of AKT, ERK1/2, and NF*κ*B. On the other hand, G*α*_12/13_/RhoA/ROCK signaling causes activation of transcription factor ATF2 to induce further proinflammatory mediator production. Finally, cytokine production, particularly IL-6, can interact with their IL receptors and promote STAT5 and STAT3 activation. In all, these pathways maintain a proinflammatory environment that leads to malignant transformation. Dashed lines denote that other proteins participate in the pathways and were omitted to summarize information. This figure is reproduced from Liu et al. [124] (under the Creative Commons Attribution License/public domain).

**Figure 4:**
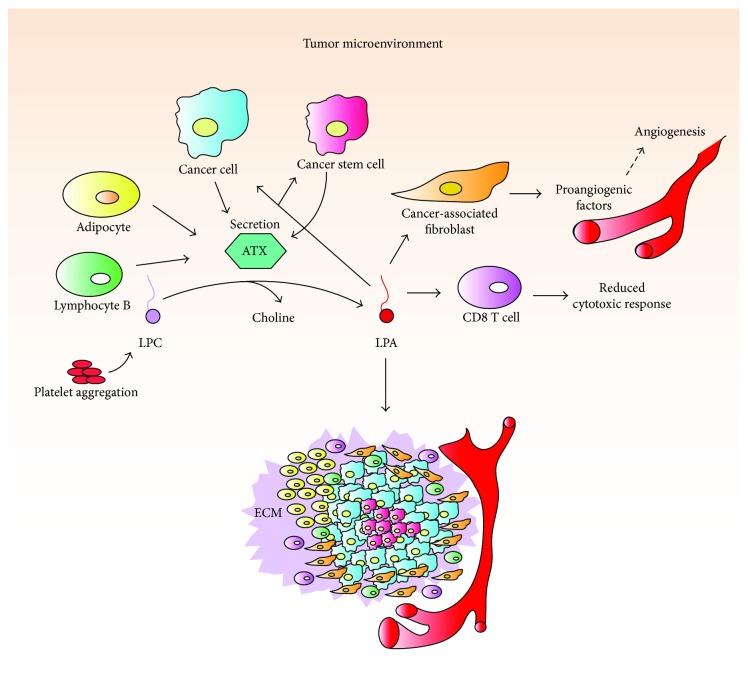
ATX-LPA signaling in tumor microenvironment. ATX hydrolyzes LPC to produce LPA from circulating LPC and platelet-derived LPC. ATX is mainly released into the tumor microenvironment by tumor-adjacent adipocytes and B lymphocytes but cancer cells and cancer stem cells also secrete this enzyme. LPA signals through its receptors to induce proliferation and invasion in cancer cells and cancer stem cells. LPA signaling induces angiogenesis through the recruitment of CAFS; it also reduces cytotoxic immune response via CD8 T cells. ECM (extracellular matrix).

So far, the evidence shows that ATX-LPA signaling in cancer is more complex than previously thought. In addition to promoting proliferation, aggressiveness, and metastasis, it induces an enabling inflammatory setting (Figure 3) and contributes to the differentiation of CAFs [123], leukocyte infiltration [92, 116], angiogenesis [123], and stem cell maintenance [99]; all of them are important components of tumor microenvironment (Figure 4). Thus, the ATX-LPA axis represents a crucial target to reduce CRI and cancer progression.

## 5. Targeting Autotaxin-LPA Axis for Cancer Therapy

LPA signaling is regulated by ATX activity, LPA receptors, and LPA degradation by LPP1 and LPAAT [125, 126]. In numerous cancers, ATX protein is overexpressed, leading to increased LPA levels in the tumor microenvironment and peripheral blood [99, 101, 127]. Cancer cells have a higher LPA receptor content on their cell surface compared to normal and benign cells and a downregulated expression of LPPs [128]. Therefore, targeting LPA signaling through these components is currently under study and constantly reviewed [4, 127, 129–132]. In this section, we summarize some of the drugs studied regarding ATX inhibition and LPA receptor antagonism (Table 1).

ATX-LPA axis has been shown to induce chemoresistance by upregulating antioxidant genes, multidrug-resistant transporters (ABCC1, ABCG2, ABCC2, and ABCC3), aldehyde dehydrogenase 1 (ALDH1), and stem cell maintenance [99, 136]. Additionally, ATX is among the top 40 most upregulated genes in metastatic cancer [146]. Therefore, inhibition of the axis has shown great results as adjuvant therapy to enhance both chemo- and radiotherapy *in vitro* and *in vivo*, as well as tumor growth reduction. Additionally, as we described, CRI is an enabling setting for tumor development. We suggest that a strategy to be considered regarding the ATX-LPA axis in CRI should be a multitarget approach, where both proinflammatory cytokines and ATX-LPA are taken into consideration for better outcomes.

Currently, drugs of ongoing clinical trials are for noncancer diseases; nevertheless, once approved, they could be tested in various cancers. Meanwhile, improvement of physiological and pathological knowledge regarding signal transduction by this axis will lead to the development of more specific therapeutic drugs to better target this signaling cascade.

## 6. Conclusions

The ATX-LPA signaling pathway is physiologically relevant during development and adulthood. Dysregulation of this axis is linked to several pathologies, including inflammation-related conditions such as rheumatoid arthritis, fibrosis, neuropathic pain, and cancer. In cancer, it has a major involvement in key components of the microenvironment, including leukocyte infiltration, angiogenesis, and decreased immune response. Interestingly, this axis has been shown to mediate cancer-related inflammation through diverse signaling pathways, crosstalk, and positive loops. Therefore, it enhances a proinflammatory microenvironment and, at the same time, ATX-LPA signaling augments. Breaking the inflammatory cycle and blocking LPA signaling and production should provide an innovative treatment for cancer by decreasing CRI, tumor growth, metastasis, and resistance to cancer treatments. Recent evidence in cirrhosis patients point to this axis as a key regulator in HCC tumorigenesis, providing a very interesting potential target for cancer prevention.

As we wait for ATX-LPA inhibitors to move from preclinical into clinical trials, further investigation is needed regarding this complex signaling pathway to achieve more efficient therapeutics in cancer and other ATX-LPA axis-related pathologies.

## Figures and Tables

**Table 1:** Targeting the ATX-LPA axis in cancer and inflammation.

Name	Target	Mechanism of action	Phase	Indication/model	Reference
HA130	ATX	It binds to the active site of ATX (T210). IC_50_ = 28 nM *in vitro*	Preclinical	Melanoma	[25]
PF-8380	ATX	Direct binding to ATX. Inhibits lysoPLD activity. IC_50_ = 2.8 nM isolated ATX IC_50_ = 101 nM *in vivo*	Preclinical	(i) Inflammation (ii) Glioblastoma	[133–135]
ONO-8430506	ATX	Direct binding to ATX. Inhibits lysoPLD activity. IC_50_ = 4.5 nM isolated ATX IC_50_ = 4.1–11.6 nM *in vivo*	Preclinical	(i) Breast cancer (ii) BCa metastasis (iii) Thyroid cancer	[19, 28, 121, 136]
GLPG1690	ATX	Binding to the hydrophobic pocket and hydrophobic channel of the protein. IC_50_ = 131 nM *in vitro*	Phase II	Idiopathic pulmonary fibrosis	[137, 138]
BMS-986020	LPA_1_	Inhibits signaling by LPA_1_	Phase II	Idiopathic pulmonary fibrosis	[139, 140]
SAR100842	LPA_1_	LPA_1_ antagonist	Phase II	Systemic sclerosis	[141]
BrP-LPA	ATX LPA_1_ LPA_2_ LPA_3_ LPA_4_ LPA_5_	Direct binding to ATX. Inhibits lysoPLD activity. IC_50_: 600 nM ex vivo Direct binding and inhibition of LPA_1–5_	Preclinical	(i) Rheumatoid arthritis (ii) Breast cancer (iii) Pancreatic cancer (iv) Glioma	[142–145]

## Abbreviations

LPA: Lysophosphatidic acid
GPCR: G protein-coupled receptor
ATX: Autotaxin
PC: Phosphatidylcholine
PS: Phosphatidylserine
PE: Phosphatidylethanolamine
PLA1: Phospholipase A1
PLA2: Phospholipase A2
LPC: Lysophosphatidylcholine
PA: Phosphatidic acid
PLD: Phospholipase D
sPLA2: Secreted phospholipase A2
LPP1: Lipid phosphate phosphohydrolase type 1
MAG: Monoacylglycerol
LPAAT: Lysophosphatidic acid acyltransferase
LPE: Lysophosphatidylethanolamine
LPS: Lysophosphatidylserine
cPLA2: Cytosolic phospholipase A2
AC: Adenylyl cyclase
EDG family: Endothelial differentiation gene family
Vgz-1: Ventricular zone gene-1
CNS: Central nervous system
COX-2: Cyclooxygenase-2
HCC: Hepatocellular carcinoma
CRI: Cancer-related inflammation
PKC: Protein kinase C
HBEpCs: Human bronchial epithelial cells
MMP: Matrix metalloprotease
Stat: Signal transducers and activators of the transcription
BCa: Breast cancer
ER: Estrogen receptor
IL: Interleukin
TNF-*α*: Tumor necrosis factor *α*
VEGF: Vascular endothelial growth factor
G-CSF: Granulocyte colony-stimulating factor
NF*κ*B: Nuclear factor kappa-light-chain-enhancer of activated B cells
ATF2: Activating transcription factor 2
OC: Ovarian cancer
ROS: Reactive oxygen species
hTERT: Human telomerase reverse transcriptase
HIF-1*α*: Hypoxia-inducible factor-1*α*
uPA: Urokinase plasminogen activator
MCP-1: Monocyte chemoattractant protein-1
MIF: Macrophage migration inhibitory factor
KLF5: Krüpple-like factor 5
FAK: Focal adhesion kinase
GBM: Glioblastoma multiforme
CAF: Cancer-associated fibroblast
EGFR: Epidermal growth factor receptor
ECM: Extracellular matrix
ALDH1: Aldehyde dehydrogenase 1.
